# Clinicopathologic characteristics and prognosis of basaloid squamous cell carcinoma of the rectum in comparison with adenocarcinoma: a retrospective cohort study

**DOI:** 10.3389/fonc.2025.1532525

**Published:** 2025-11-11

**Authors:** Yanyang Zhou, Yi Chang, Haixia Guo, Yanwei Fan, Shijie Wang

**Affiliations:** 1 Anorectal Department, The First Affiliated Hospital of Henan University of Chinese Medicine, Zhengzhou, China; 2 College of Oral Health, Zhengzhou Health College, Zhengzhou Shuqing Medical College, Zhengzhou, China; 3 Department of General Surgery, Jinling Hospital, Affiliated Hospital of Medical School, Nanjing University, Nanjing, China

**Keywords:** China basaloid squamous cell carcinoma, rectum, prognosis, SEER, adenocarcinoma

## Abstract

**Objective:**

Basaloid squamous cell carcinoma (BSCC) is a rare histological subtype of rectal cancer. This study aimed to investigate the clinicopathologic features and survival outcomes of primary rectal BSCC and to compare them with those of adenocarcinoma (AD).

**Methods:**

Patients diagnosed with rectal BSCC and AD patients were identified from the Surveillance, Epidemiology, and End Results database from 2000 to 2019. Univariable and multivariable Cox regression analyses were performed to identify potential risk factors for survival in BSCC patients. A nomogram was constructed to predict the prognosis of BSCC patients.

**Results:**

A total of 257 BSCC and 77,094 AD patients were identified. BSCC patients were more significantly correlated with female gender, early clinical stage, and poor differentiation. Significantly better 5-year overall survival (OS) and cancer-specific survival (CSS) were observed in patients with BSCC than those with AD (OS: 60.4% *vs*. 52.0%, *p* = 0.041; CSS: 63.4% *vs*. 54.5%, *p* = 0.042). This survival difference still persisted in multivariable analysis [OS: hazard ratio (HR) = 0.269, *p* < 0.001] and after propensity score matching (OS: HR = 0.387, *p* < 0.001). For BSCC patients, multivariable analysis indicated that advanced clinical stage was associated with worse OS and CSS and that surgery and chemotherapy were associated with better OS and CSS.

**Conclusion:**

Patients with rectal BSCC have significantly better survival outcomes than those with rectal AD, with a more early clinical stage. Surgery in combination with chemotherapy is the preferred treatment to improve prognosis.

## Introduction

Colorectal cancer ranks second in mortality rate and third in incidence among malignant tumors worldwide, and approximately 30% of these cases are found in the rectum (1). Determining the ideal treatment plans for rectal cancer is a multifaceted process, in which the pathological type is the most important factor (2). Although adenocarcinomas account for more than 90% of rectal cancers, other rare pathological types, such as basaloid squamous cell carcinoma (BSCC), cannot be ignored in clinical practice (3). The pathological features of BSCC include a palisading pattern, scant cytoplasm, and elongated nuclei, and formal diagnosis usually requires immunohistochemical staining (4). BSCC is an aggressive variant of squamous cell carcinoma (SCC), characterized by rapid disease progression and a high tendency for distant metastasis (4). The most common sites of BSCC are the upper digestive tract (including the oropharynx), lungs, and anal canal (4, 5), while rectal BSCC is so rare that its clinical prognosis and optimal treatment strategies remain unknown. To our knowledge, only several case reports have been published about rectal BSCC (4, 6–9). Therefore, the objective of this study was to investigate the clinicopathologic features and survival outcomes of primary rectal BSCC and to compare them with those of adenocarcinoma (AD).

## Method

### Data source and study cohort

The Surveillance, Epidemiology, and End Results (SEER) database is the largest publicly available cancer database, accounting for approximately 48% of the US population. Patients were identified and selected from the SEER database, and individual patient-level data were retrieved using SEER*Stat Version 8.4.0.1 (https://seer.cancer.gov/seerstat/).

The data of patients diagnosed with BSCC and AD were collected retrospectively during the period from 2000 to 2019. The International Classification of Diseases for Oncology, 3rd edition (ICD-O-3), was utilized to identify primary sites using code C20.9. The histological types of BSCC were defined using histology codes 8083/3 and those of AD using codes 8140/3, 8141/3, 8144/3, 8210/3, 8211/3, 8221/3, and 8261/3–8263/3. Patients who were lost to follow-up, had missing key clinical information, had non-primary tumors, or lacked pathological confirmation were excluded. The primary survival outcomes were overall survival (OS) and cancer-specific survival (CSS).

### Statistical analysis

Schoenfeld residuals were employed to assess the proportional hazards (PH) assumption, and the results demonstrated that the PH assumption was satisfied (*p* = 0.079). The TNM stages were standardized according to the 7th edition TNM staging criteria based on tumor invasion depth, number of positive lymph nodes, and distant metastasis status. The categorical variables of clinicopathologic characteristics were compared using the chi-square test and Fisher’s exact test. The survival curves were depicted using the Kaplan–Meier method, and the log-rank test was used to compare differences. Independent risk factors were identified using the multivariable Cox proportional hazards model. Furthermore, propensity score matching (PSM) was implemented to balance the confounding baseline variables between the BSCC and AD groups. A nomogram based on the results of multivariable analysis and clinical experience was constructed to estimate the prognosis of patients with rectal BSCC. The C-index and receiver operating characteristic (ROC) were used to evaluate the accuracy of the nomogram, and the calibration plots were depicted to verify the discrimination of the nomogram. A two-sided *p*-value < 0.05 was considered statistically significant. Data analysis was conducted utilizing the SPSS software version 28.0 (IBM Corporation, USA) and R Version 4.1.3 (R Foundation for Statistical Computing, Austria).

## Results

### Demographic and clinicopathologic characteristics

**Figure 1:**
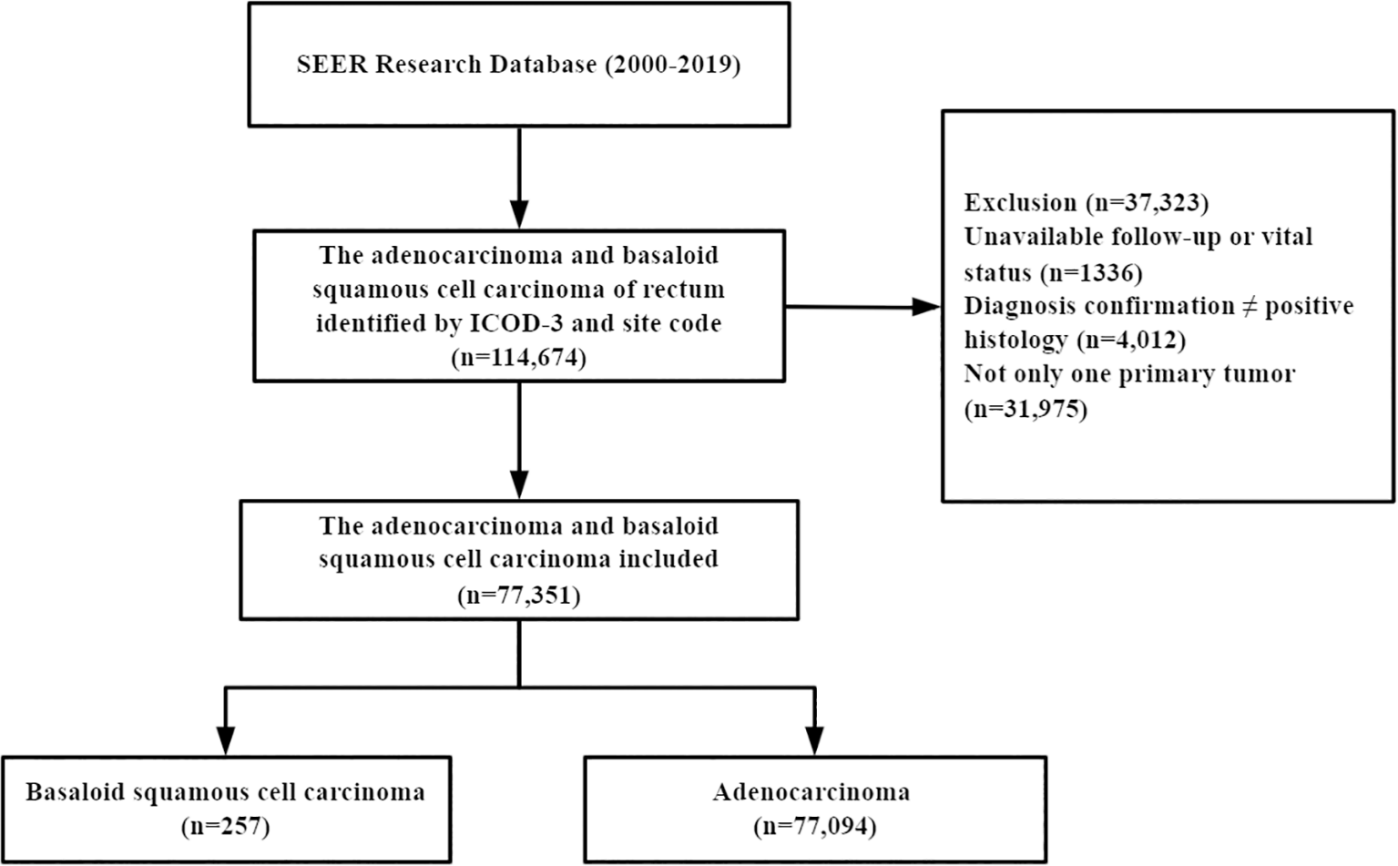
Flow diagram of patient selection.

A total of 77,351 patients were identified from the SEER database, of which 257 (0.33%) had BSCC and 77,094 (99.67%) had AD (**Figure 1**). The baseline and clinicopathologic characteristics are displayed in **Table 1** for the entire cohort. The mean age of the patients with BSCC was 63.1 years, with a male-to-female ratio of 0.37. Of the patients, 47.5% were at stages I–II at initial diagnosis, and 44.4% presented with poorly/undifferentiated cancer.

**Table 1:** Baseline demographic and clinicopathologic characteristics of patients with BSCC of the rectum compared to AD.

Characteristic	BSCC (n = 257)	AD (n = 77,094)	*p*-Value
Age			
<65	136 (52.9%)	41,675 (54.1%)	0.715
≥65	121 (47.1%)	35,419 (45.9%)	
Sex			
Male	69 (26.8%)	45,888 (59.5%)	<0.001
Female	188 (73.2%)	31,206 (40.5%)	
Race			
White	224 (87.2%)	62,456 (81.0%)	<0.001
Black	26 (10.1%)	6,756 (8.8%)	
Other	7 (2.7%)	7,882 (10.2%)	
Clinical T-stage			
T1–T2	191 (74.3%)	34,690 (45.4%)	<0.001
T3–T4	66 (25.7%)	41,695 (54.6%)	
Unknown	0 (0%)	709 (0.9%)	
Clinical N-stage			
N0	151 (58.8%)	40,853 (53.0%)	<0.001
N1/N2	46 (17.9%)	26,064 (33.8%)	
Unknown	60 (23.3%)	10,177 (13.2%)	
Clinical M-stage			
M0	189 (73.5%)	59,554 (77.2%)	<0.001
M1	27 (10.5%)	14,944 (19.4%)	
Unknown	41 (16.0%)	2,596 (3.4%)	
AJCC clinical stage			
I + II	122 (47.5%)	33,829 (43.9%)	<0.001
III + IV	62 (24.1%)	36,868 (47.8%)	
Unknown	73 (28.4%)	6,397 (8.3%)	
Histological grade			
Well/moderately differentiated	18 (7.0%)	54,628 (70.9%)	<0.001
Poorly/undifferentiated	114 (44.4%)	9,007 (11.7%)	
Unknown	125 (48.6%)	13,459 (17.5%)	
Surgery			
Yes	67 (26.1%)	56,884 (73.8%)	<0.001
No	190 (73.9%)	20,210 (26.2%)	
Radiotherapy			
Yes	51 (19.8%)	34,246 (44.4%)	<0.001
No	206 (80.2%)	42,848 (55.6%)	
Chemotherapy			
Yes	165 (64.2%)	47,862 (62.1%)	0.484
No	92 (35.8%)	29,232 (37.9%)	
Year of diagnosis			
2000–2009	109 (42.4%)	38,938 (50.5%)	0.01
2010–2019	148 (57.6%)	38,156 (49.5%)	
Marital status			
Married	129 (50.2%)	44,038 (57.1%)	0.025
Unmarried^a^	128 (49.8%)	33,056 (42.9%)	
Survival status			
Alive	144 (56.0%)	35,754 (46.4%)	0.002
Dead	113 (44.0%)	41,340 (53.6%)	

BSCC, basaloid squamous cell carcinoma; AD, adenocarcinoma; T, tumor; N, node; M, metastasis.

a Unmarried included single, divorced, widowed, and separated.

Significant differences were observed in BSCC patients when compared with AD patients, who were more often female, had an early clinical stage, had poor differentiation, and were unmarried; fewer patients received surgery and radiotherapy.

### Survival

**Figure 2:**
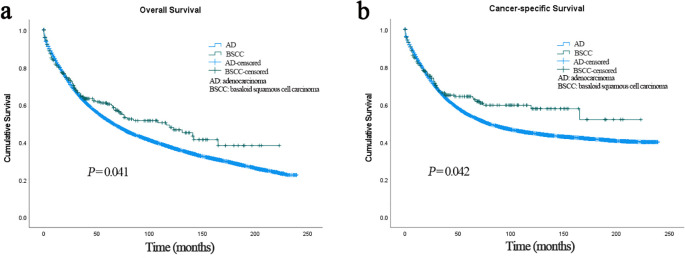
**(a)** Overall survival of patients with BSCC and AD. **(b)** Cancer-specific survival of patients with BSCC and AD. BSCC, basaloid squamous cell carcinoma; AD, adenocarcinoma.

In the BSCC group, the median follow-up time was 38 months, and death occurred in 113 (44.0%) patients. In the AD group, the median follow-up time was 35 months, and death occurred in 41,340 (53.6%) patients. Patients with BSCC had significantly higher 3-, 5-, and 10-year OS and CSS than those with AD (OS: 64.3%, 60.4%, and 48.0% *vs*. 63.3%, 52.0%, and 37.4%, *p* = 0.041; CSS: 66.2%, 63.4%, and 57.8% *vs*. 64.8%, 54.5%, and 44.8%, *p* = 0.042) (**Figure 2**). Histological type was identified as an independent prognostic factor, with BSCC associated with significantly better OS and CSS than AD [OS: hazard ratio (HR) = 0.269, 95% confidence interval (CI) = 0.191–0.379; CSS: HR = 0.258, 95% CI = 0.175–0.379] (**Table 2**). To match the basic characteristics, PSM analysis was performed (**Supplementary Table S1**). After matching, a total of 101 BSCC patients and 195 AD patients were included, with no significant differences in baseline variables between the two groups except for year of diagnosis. Further multivariable analysis of the matched cohort showed that BSCC remained an independent favorable predictor of OS (HR = 0.387, 95% CI = 0.248–0.602) (**Supplementary Table S2**).

**Table 2:** Univariate analysis and multivariable analysis of entire cohort.

Characteristic	Overall survival				Cancer-specific survival			
	Univariate analysis		Multivariable analysis		Univariate analysis		Multivariable analysis	
	HR (95% CI)	*p*-Value	HR (95% CI)	*p*-Value	HR (95% CI)	*p*-Value	HR (95% CI)	*p*-Value
Age								
<65	Reference	–	Reference	–	Reference	–	Reference	–
≥65	2.113 (2.072–2.155)	<0.001	1.974 (1.929–2.019)	<0.001	2.043 (1.997–2.091)	<0.001	1.853 (1.804–1.904)	<0.001
Sex								
Male	Reference	–	–	–	Reference	–	–	–
Female	0.993 (0.974–1.013)	0.496	–	–	0.995 (0.972–1.018)	0.648	–	–
Race								
White	Reference	–	Reference	–	Reference	–	Reference	–
Black	1.287 (1.246–1.329)	<0.001	1.177 (1.133–1.223)	<0.001	1.361 (1.312–1.413)	<0.001	1.182 (1.132–1.236)	<0.001
Other	0.867 (0.838–0.897)	<0.001	0.875 (0.842–0.910)	<0.001	0.889 (0.854–0.924)	<0.001	0.890 (0.851–0.930)	<0.001
Clinical T-stage								
T1–2	Reference	–	–	–	Reference	–	–	–
T3–4	1.113 (1.092–1.135)	<0.001	–	–	1.10 (1.08–1.13)	<0.001	–	
Clinical N-stage								
N0	Reference	–	–	–	Reference	–	–	–
N1/N2	1.236 (1.209–1.264)	<0.001			1.351 (1.314–1.389)	<0.001	–	
Clinical M-stage								
M0	Reference	–	–	–	Reference	–	–	–
M1	4.590 (4.487–4.694)	<0.001			5.561 (5.415–5.711)	<0.001	–	
AJCC clinical stage								
I + II	Reference	–	Reference	–	Reference	–	Reference	–
III + IV	2.001 (1.960–2.044)	<0.001	2.200 (2.146–2.256)	<0.001	2.864 (2.788–2.942)	<0.001	2.884 (2.794–2.976)	<0.001
Surgery								
No	Reference	–	Reference	–	Reference	–	Reference	–
Yes	0.247 (0.242–0.252)	<0.001	0.289 (0.280–0.299)	<0.001	0.203 (0.199–0.208)	<0.001	0.253 (0.244–0.262)	<0.001
Radiation								
No	Reference	–	Reference		Reference	–	Reference	–
Yes	0.465 (0.456–0.475)	<0.001	0.857 (0.829–0.886)	<0.001	0.434 (0.424–0.445)	<0.001	0.880 (0.847–0.914)	<0.001
Chemotherapy								
No	Reference	–	Reference	–	Reference	–	Reference	–
Yes	0.732 (0.718–0.747)	<0.001	0.686 (0.665–0.708)	<0.001	0.791 (0.773–0.810)	<0.001	0.673 (0.650–0.698)	<0.001
Differentiated grade								
Well/moderately differentiated	Reference	–	Reference	–	Reference	–	Reference	–
Poorly/undifferentiated	1.451 (1.411–1.492)	<0.001	1.424 (1.383–1.466)	<0.001	1.648 (1.597–1.700)	<0.001	1.553 (1.502–1.605)	<0.001
Histology								
AD	Reference	–	Reference	–	Reference	–	Reference	–
BSCC	0.825 (0.686–0.992)	0.041	0.269 (0.191–0.379)	<0.001	0.793 (0.634–0.992)	0.042	0.258 (0.175–0.379)	<0.001
Year of diagnosis								
2000–2009	Reference	–	Reference	–	Reference	–	Reference	–
2010–2019	0.892 (0.873–0.911)	<0.001	0.816 (0.796–0.836)	<0.001	0.750 (0.732–0.768)	<0.001	0.650 (0.632–0.668)	<0.001
Marital status								
Unmarried^a^	Reference	–	Reference	–	Reference	–	Reference	–
Married	1.603 (1.572–1.634)	<0.001	1.325 (1.295–1.355)	<0.001	1.661 (1.623–1.699)	<0.001	1.331 (1.296–1.368)	<0.001

BSCC, basaloid squamous cell carcinoma; AD, adenocarcinoma; T, tumor size; M, metastasis; N, node; HR, hazard ratio; CI, confidence interval.

a Unmarried included single, divorced, widowed, and separated.

The prognostic factors of the 257 patients with BSCC were further analyzed (**Table 3**). In univariate analysis, age, gender, M stage, American Joint Committee on Cancer (AJCC) clinical stage, surgery, chemotherapy, radiotherapy, and married status were significantly associated with OS and CSS. In multivariable analysis, advanced stage and unmarried status were identified as independent risk factors for OS and CSS, and surgery and chemotherapy were significantly associated with better OS and CSS.

**Table 3:** Univariate analysis and multivariable analysis of BSCC.

Characteristic	Overall survival				Cancer-specific survival			
	Univariate analysis		Multivariable analysis		Univariate analysis		Multivariable analysis	
	HR (95% CI)	*p*-Value	HR (95% CI)	*p*-Value	HR (95% CI)	*p*-Value	HR (95% CI)	*p*-Value
Age								
<65	Reference	–	Reference	–	Reference	–	Reference	–
≥65	1.683 (1.159–2.445)	0.006	1.516 (0.934–2.461)	0.092	1.880 (1.186–2.981)	0.007	1.529 (0.852–2.743)	0.154
Sex								
Male	Reference	–	Reference	–	Reference	–	Reference	–
Female	0.667 (0.447–0.995)	0.047	0.649 (0.384–1.094)	0.105	0.595 (0.364–0.971)	0.038	0.682 (0.358–1.301)	0.246
Race								
White	Reference	–	Reference	–	Reference	–	–	–
Black	1.202 (0.685–2.109)	0.521	1.434 (0.690–2.979)	0.334	1.384 (0.710–2.699)	0.34	–	–
Other	12.107 (2.843–51.557)	< 0.001	19.320 (4.026-92.717)	< 0.001	–	–	–	–
Clinical T-stage								
T1–2	Reference	–	–	–	Reference	–	–	–
T3–4	1.598 (1.070–2.387)	0.022	–	–	1.566 (0.965–2.542)	0.069	–	–
Clinical N-stage								
N0	Reference	–	–	–	Reference	–	–	–
N1/N2	1.266 (0.747–2.145)	0.38	–	–	1.523 (0.838–2.769)	0.168	–	–
Clinical M-stage								
M0	Reference	–	–	–	Reference	–	–	–
M1	5.418 (3.363–8.728)	< 0.001	–	–	7.315 (4.242–12.614)	< 0.001	–	–
AJCC clinical stage								
I + II	Reference	–	Reference	–	Reference	–	Reference	–
III + IV	2.073 (1.303–3.298)	0.002	2.027 (1.233–3.331)	0.005	3.123 (1.771–5.506)	<0.001	3.220 (1.755–5.910)	< 0.001
Surgery								
No	Reference	–	Reference	–	Reference	–	Reference	–
Yes	0.376 (0.224–0.631)	< 0.001	0.294 (0.103–0.842)	0.023	0.305 (0.152–0.613)	< 0.001	0.268 (0.078–0.918)	0.036
Radiation								
No	Reference	–	Reference		Reference	–	Reference	–
Yes	0.298 (0.155–0.571)	< 0.001	1.127 (0.351–3.625)	0.841	0.183 (0.067–0.502)	< 0.001	1.062 (0.249–4.522)	0.935
Chemotherapy								
No	Reference	–	Reference	–	Reference	–	Reference	–
Yes	0.540 (0.372–0.782)	0.001	0.460 (0.273–0.774)	0.003	0.411 (0.260–0.649)	< 0.001	0.329 (0.177–0.612)	< 0.001
Differentiated grade								
Well/moderately differentiated	Reference	–	–	–	Reference	–	–	–
Poorly/undifferentiated	0.615 (0.317–1.194)	0.151	–	–	0.533 (0.252–1.127)	0.103	–	–
Year of diagnosis								
2000–2009	Reference	–	–	–	Reference	–	–	–
2010–2019	0.830 (0.563–1.226)	0.349	–	–	0.685 (0.430–1.091)	0.111	–	–
Marital status								
Married	Reference	–	Reference	–	Reference	–	Reference	–
Unmarried^a^	2.153 (1.435–3.231)	< 0.001	1.889 (1.135–3.144)	0.014	2.354 (1.438–3.852)	<0.001	2.033 (1.098–3.765)	0.024

BSCC, basaloid squamous cell carcinoma; AD, adenocarcinoma; T, tumor size; M, metastasis; N, node; HR, hazard ratio; CI, confidence interval.

a Unmarried included single, divorced, widowed, and separated.

**Figure 3:**
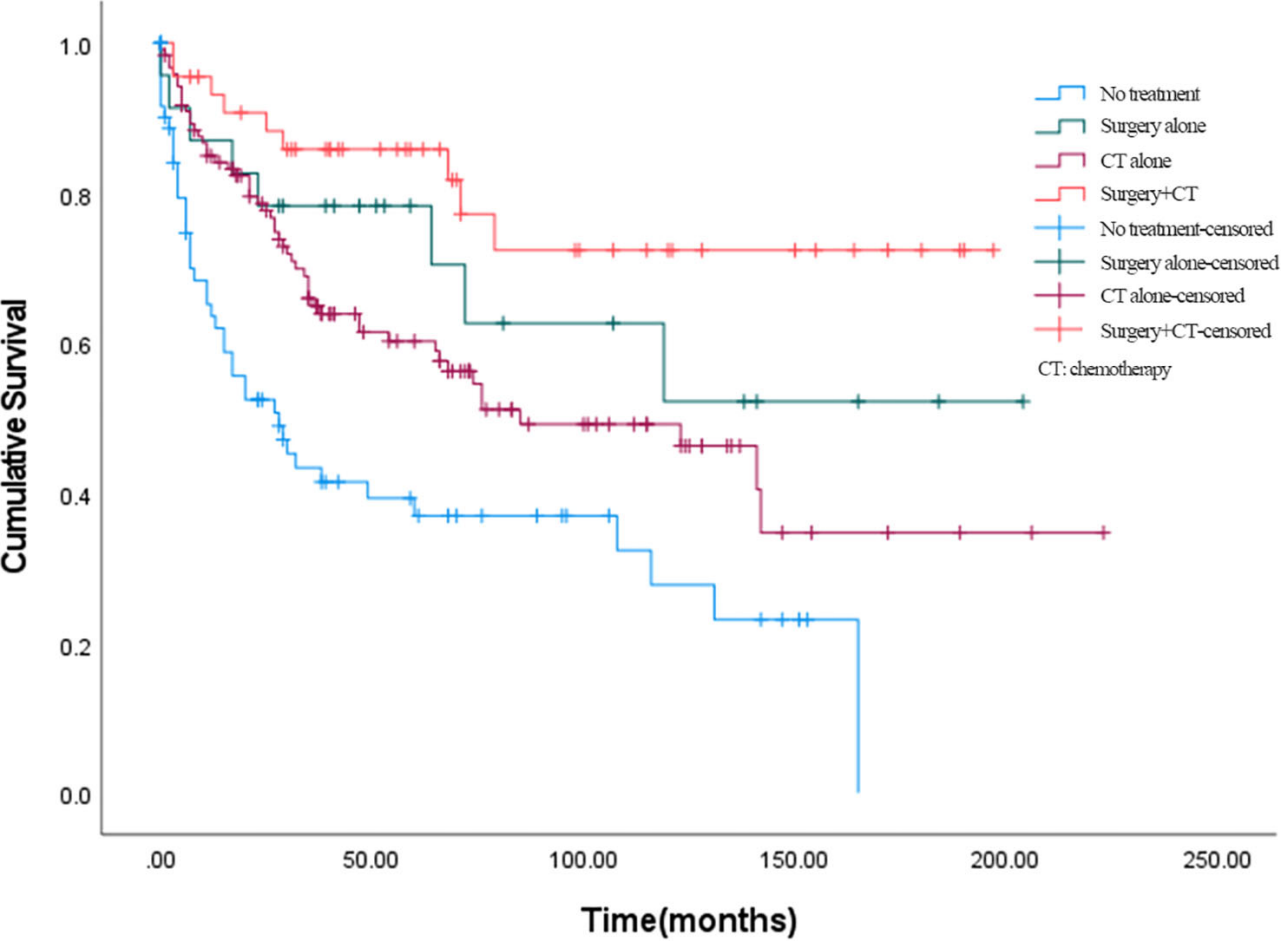
Effect of treatment modalities on overall survival. CT, chemotherapy.

The effects of different treatment modalities for BSCC patients were further explored. In all enrolled patients with BSCC, 23 (8.9%) received surgery alone, 119 (46.3%) received chemotherapy alone, 44 (17.1%) received surgery plus chemotherapy, and 71 (27.6%) received no treatment; the number of deaths corresponding to the above treatment modalities was 8, 53, 9, and 28 cases, respectively. Among them, 45 people received chemoradiotherapy. The 5-year OS rates of patients who received surgery alone, chemotherapy alone, surgery plus chemotherapy, or no treatment were 70.4%, 58.9%, 81.7%, and 37.0%, respectively (**Figure 3**). Overall, combination therapy (surgery plus chemotherapy) showed a lower hazard ratio (HR = 0.196, 95% CI = 0.095–0.403) compared to surgery alone (HR = 0.350, 95% CI = 0.164–0.746) or chemotherapy alone (HR = 0.516, 95% CI = 0.345–0.772).

### Prognostic nomogram

**Figure 4:**
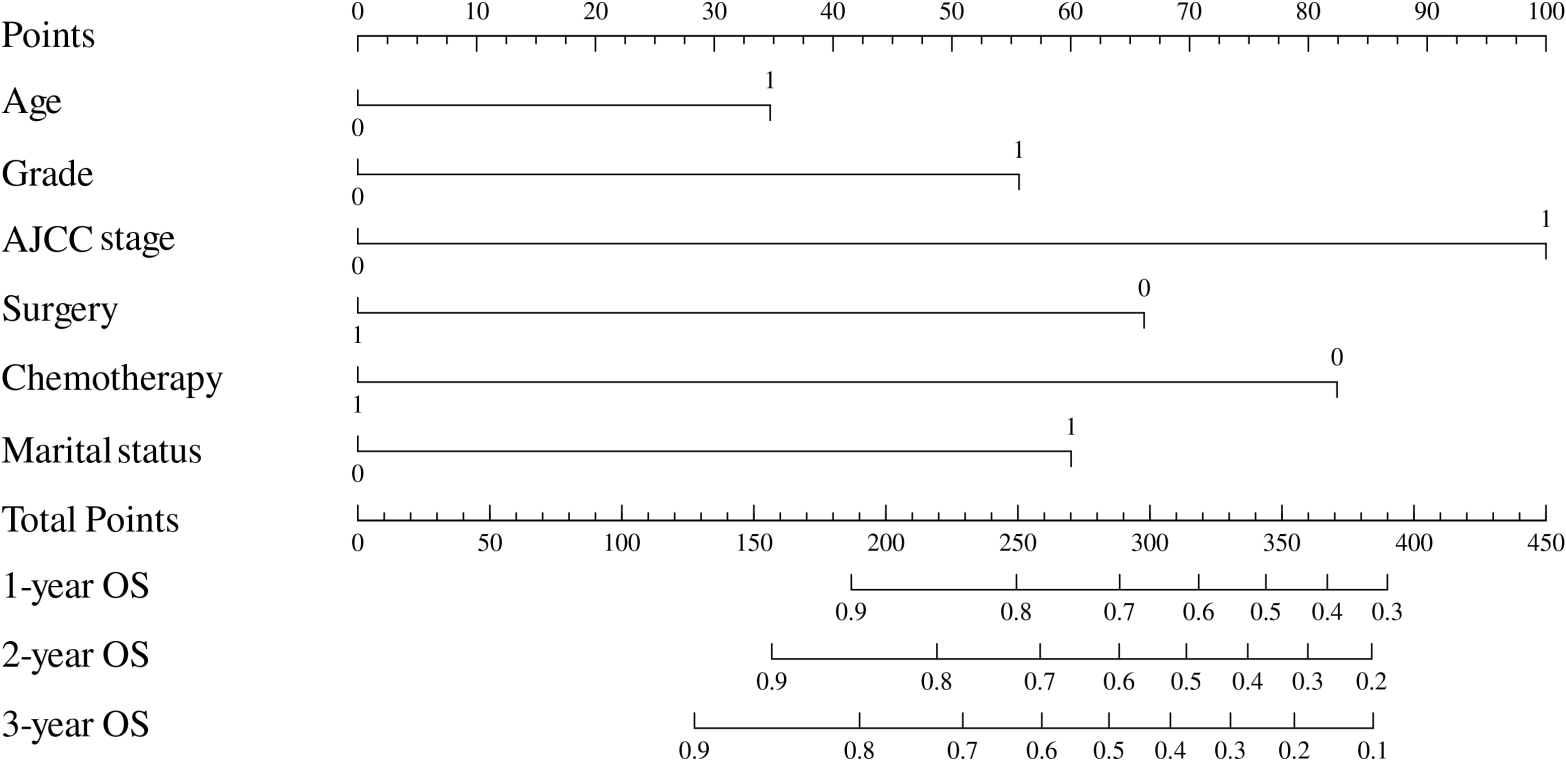
Nomogram to estimate the prognosis of patients with rectal BSCC. BSCC, basaloid squamous cell carcinoma.

**Figure 5:**
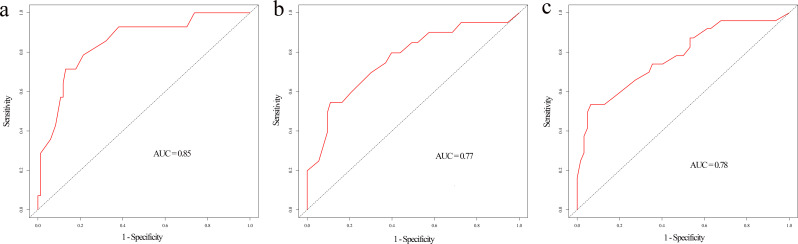
Time-dependent ROC curve of the nomogram at 1-year **(a)**, 2-year **(b)**, and 3-year **(c)** overall survival.

Based on the results of multivariable analysis and clinical experience, age, differentiated grade, AJCC stage, surgery, chemotherapy, and marital status were included to establish a prognostic nomogram for patients with BSCC (**Figure 4**). In the time-dependent ROC analysis, the nomogram showed a good ability to identify 1-, 2-, and 3-year OS, with area under the curve (AUCs) of 0.85 (0.74–0.96), 0.77 (0.64–0.89), and 0.78 (0.66–0.89), respectively (**Figure 5**). Internal validation was performed using 1,000 bootstrap analyses; their corrected C-indexes were 0.84, 0.74, and 0.76, respectively. Meanwhile, the calibration plot of the nomogram showed a good consistency between the predicted survival and actual observations (**Supplementary Figure S1**).

## Discussion

Due to the rarity of primary rectal BSCC, its clinicopathologic characteristics and prognosis remain poorly understood. The current knowledge of rectal BSCC is sourced only from sporadic case reports, with no definitive understanding (4, 6–9). Therefore, it is essential to utilize a large database such as SEER for a larger sample size and more comprehensive analyses. To our knowledge, we conducted the first population-based cohort study investigating the clinicopathologic features, survival, and treatment specific to rectal BSCC.

BSCC presents its unique histopathological features, composed of two types of tumor cells, basaloid and squamous cells (10). Although the upper aerodigestive tract is the most common site, some rare sites, such as the lungs, colon, and rectum, are occasionally found (4, 11). Previous studies have reported survival trends of BSCC; most have focused on BSCC of the head and neck or esophagus. In contrast, our research specifically focused on rectal BSCC (4, 5). In this study, we enrolled 257 patients with BSCC; the mean age of BSCC patients was 63.1 years, similar to patients with AD. However, contrary to the known male predilection of rectal AD (12), our study revealed a male-to-female ratio of 0.37 for BSCC, presenting a female predilection. Meanwhile, our study showed that patients with BSCC were more significantly associated with early clinical stage and poor differentiation. After we adjusted for potential clinical and demographical confounding factors, BSCC was identified as an independent, favorable prognostic factor for survival. Basaloid squamous cell carcinoma lacks typical squamous morphology but exhibits immunohistochemical expression of squamous markers, with positive p63 staining localized in cell nuclei and superficial regions (11, 13). Increased expression of p53 and Ki-67 is often associated with poorer prognosis. Previous studies have reported that the positive staining rates of p53 and Ki-67 in BSCC are 55.0% and 57.5%, respectively, compared with 85.4% and 75.6% in colorectal adenocarcinoma (14, 15). This difference may partly explain the finding that BSCC has better survival than adenocarcinoma. However, current studies still show inconsistent results regarding the prognosis of BSCC patients. For instance, in the lung, BSCC has been found to have a better prognosis than AD, which is consistent with our results (11). In contrast, a previous study reported that colorectal BSCC may have a poorer prognosis than AD (4). Notably, this study included only 10 patients without a control cohort, which may have introduced selection bias. Thus, future verification with large-scale cohort studies is still needed.

Given the significant differences in tumor characteristics between BSCC and AD, further exploration of prognostic factors specific to BSCC was necessary. Advanced age has long been recognized as a poor prognostic factor for most known malignancies, probably because of the higher incidence of comorbidities (16, 17). In our study, age > 65 was also determined to be an independent prognostic factor of poor OS and CSS. Just as previous studies have reported that tumor stage is an important prognostic factor for rectal cancer (18), our study also found that advanced stage was significantly associated with worse OS and CSS. After excluding patients with missing stage information, stage III–IV tumors accounted for 33.7% and 52.1% of BSCC and AD, respectively, which may partly explain the better prognosis of BSCC patients. Interestingly, our study found that married status was an independent prognostic factor for patients with BSCC; a similar phenomenon was found for many cancers, such as cervical, pancreatic, and lung cancers (19–21). Several hypotheses have been proposed to explain the poorer prognosis in unmarried patients, such as financial support and assistance with daily living (21).

Notably, prior to our work, there was a lack of clear understanding of optimal treatment strategies for this rare disease. The clinical decisions were often made based solely on extrapolated experience from rectal AD. The current standard approach to treating rectal cancer is total tumor excision with adjuvant chemoradiotherapy (22). However, due to the limited number of cases and studies on rectal BSCC, it remains unclear whether this strategy is the optimal treatment modality. Among the five patients with rectal BSCC previously reported, one received surgery alone, one received surgery plus chemotherapy, and three patients received surgery plus chemoradiotherapy (4, 6–9). With the exception of one patient who was lost to follow-up, patients with surgery combined with chemotherapy had the longest survival at 96 months (4). Similarly, in our study, we also found that surgery plus chemotherapy improved patient outcomes better than surgery alone or chemotherapy alone. As for radiotherapy, the prognostic benefit was not observed in our study, probably due to the limited number of patients who received radiotherapy. However, a multicenter randomized controlled study demonstrated that preoperative adjuvant radiotherapy reduces the recurrence rate of rectal cancer after surgery (23). In addition, the French Society of Oncological Radiotherapy also recommends that patients with advanced rectal cancer should be treated with postoperative chemoradiotherapy (24). For BSCC, a special pathological type, radiotherapy has also been reported to improve the prognosis of patients with laryngopharyngeal BSCC (25). Therefore, the importance of radiotherapy in the management of patients with rectal BSCC cannot be ignored.

This study is the first and largest study of rectal BSCC to date, but some limitations still exist. First, due to the retrospective study design, selection bias could not be avoided. In addition, the limited number of patients precluded clinical stage-based subgroup analyses, and the lack of external validation limited the generalizability of the conclusions. Third, several other factors that may affect survival, such as surgical margins and perineural invasion, were not accessible in the SEER database. Finally, due to the small number of BSCC patients who received radiotherapy, the role of radiotherapy in these patients was not fully validated, and other treatment strategies also require further validation. Given the above limitations, large-scale prospective cohort studies are needed to better validate the conclusions of this study in the future.

## Conclusion

Rectal BSCC is an extremely rare histological subtype of rectal cancer with a significantly better prognosis than rectal AD. For rectal BSCC patients, advanced stage and unmarried status were significantly associated with worse OS and CSS. Surgery alone and chemotherapy alone can significantly improve patient survival, and surgery combined with chemotherapy is the preferred treatment.

## Data Availability

The datasets presented in this study can be found in online repositories. The names of the repository/repositories and accession number(s) can be found below: https://seer.cancer.gov/data/.

## Supplementary material

Supplementary Figure 1 Calibration curve of the nomogram at 1-year (a), 2-year (b) and 3-year (c) overall survival.
