# Supplementary figures and images for: Clinicopathologic characteristics and prognosis of basaloid squamous cell carcinoma of the rectum in comparison with adenocarcinoma: a retrospective cohort study
Source: Front Oncol. 2025 Nov 11;15:1532525. doi: 10.3389/fonc.2025.1532525 (PMC12643862; doi:10.3389/fonc.2025.1532525)

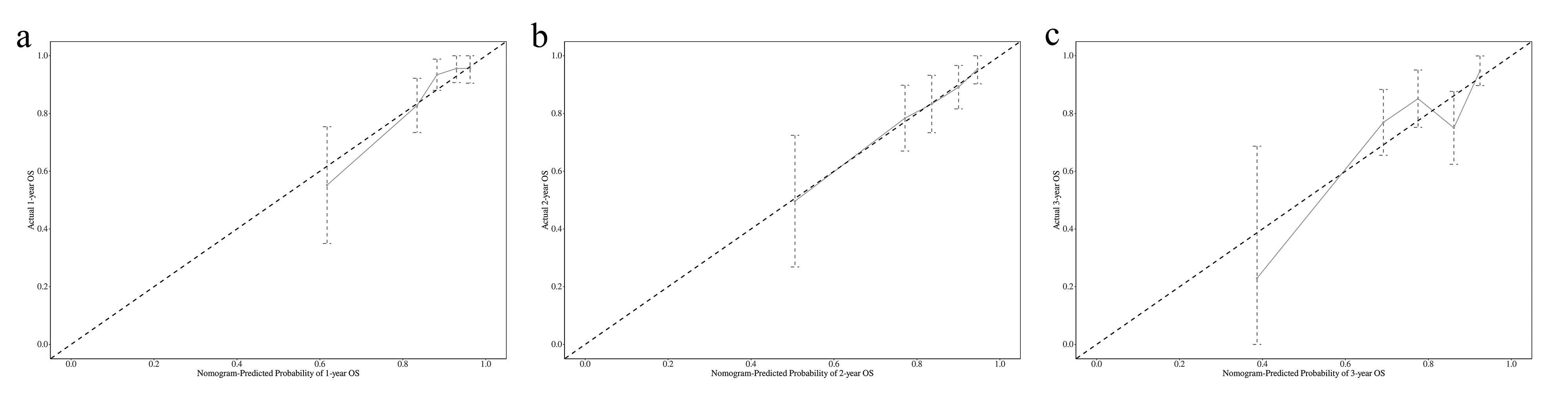

Supplement: Supplementary Figure 1 — Calibration curve of the nomogram at 1-year (a), 2-year (b) and 3-year (c) overall survival. [file Image1.tif]
