# Supplementary material for: Clinicopathologic characteristics and prognosis of basaloid squamous cell carcinoma of the rectum in comparison with adenocarcinoma: a retrospective cohort study
Source: Front Oncol. 2025 Nov 11;15:1532525. doi: 10.3389/fonc.2025.1532525 (PMC12643862; doi:10.3389/fonc.2025.1532525)
Supplement: Supplementary file 2 [file Table1.docx]

Table S1: Patient characteristics after propensity score matching

|  | **BSCC (n=** **101)** | **AD (n=** **195)** | ***P* Value** | **\| SMD \|** |
| --- | --- | --- | --- | --- |
| Age |  |  |  |  |
| < 65 | 51 (50.5%) | 97 (49.7%) | 0.902 | 0.015 |
| ≥ 65 | 50 (49.5%) | 98 (50.3%) |  |  |
| Sex |  |  |  |  |
| Male | 24 (23.8%) | 48 (24.6%) | 0.871 | 0.02 |
| Female | 77 (76.2%) | 147 (75.4%) |  |  |
| Race |  |  |  |  |
| White | 89 (88.1%) | 173 (88.7%) | 0.930 | 0.019 |
| Black | 11 (10.9%) | 18 (9.2%) |  |  |
| Other | 1 (1.0%) | 2 (1.0%) |  |  |
| AJCC Clinical stage |  |  |  |  |
| I + II | 65 (64.4%) | 123 (63.1%) | 0.828 | 0.027 |
| III + IV | 36 (35.6%) | 72 (36.9%) |  |  |
| Histologic grade |  |  |  |  |
| Well/Moderately differentiated | 11 (10.9%) | 22 (11.3%) | 0.919 | 0.012 |
| Poorly/Undifferentiated | 90 (89.1%) | 173 (88.7%) |  |  |
| Surgery |  |  |  |  |
| Yes | 65 (64.4%) | 123 (63.1%) | 0.828 | 0.027 |
| No | 36 (35.6%) | 72 (36.9%) |  |  |
| Radiotherapy |  |  |  |  |
| Yes | 28 (27.7%) | 56 (28.7%) | 0.857 | 0.022 |
| No | 73 (72.3%) | 139 (71.3%) |  |  |
| Chemotherapy |  |  |  |  |
| Yes | 28 (27.7%) | 54 (27.7%) | 0.996 | 0.001 |
| No | 73 (72.3%) | 141 (72.3%) |  |  |
| Year of diagnosis |  |  |  |  |
| 2000-2009 | 40 (39.6%) | 182 (93.3%) | <0.001 | 2.154 |
| 2010-2019 | 61 (60.4%) | 13 (6.7%) |  |  |
| Marital status |  |  |  |  |
| Married | 50 (49.5%) | 85 (43.6%) | 0.333 | 0.119 |
| Unmarried^a^ | 51 (50.5%) | 110 (56.4%) |  |  |

BSCC, basaloid squamous cell carcinoma; AD, adenocarcinoma; ^a^ Unmarried included single, divorced, widowed, and separated;
