# Supplementary material for: Clinicopathologic characteristics and prognosis of basaloid squamous cell carcinoma of the rectum in comparison with adenocarcinoma: a retrospective cohort study
Source: Front Oncol. 2025 Nov 11;15:1532525. doi: 10.3389/fonc.2025.1532525 (PMC12643862; doi:10.3389/fonc.2025.1532525)
Supplement: Supplementary file 3 [file Table2.docx]

Table S2: Univariate Analysis and Multivariate Analysis for Entire Cohort after PM

| Characteristic | Univariate Analysis | | Multivariate Analysis | |
| --- | --- | --- | --- | --- |
|  | HR (95% CI) | *P* Value | HR (95% CI) | *P* Value |
| Age |  |  |  |  |
| ＜65 | Reference | - | Reference | - |
| ≥65 | 1.473 (1.107 - 1.960) | 0.008 | 1.693 (1.236 - 2.319) | 0.001 |
| Sex |  |  |  |  |
| Male | Reference | - | - | - |
| Female | 0.878 (0.636 - 1.211) | 0.428 | - | - |
| Race |  |  |  |  |
| White | Reference | - | - | - |
| Black | 1.223 (0.783 - 1.908) | 0.376 | - | - |
| Other | 0.883 (0.219 - 3.565) | 0.861 | - | - |
| AJCC Clinical stage |  |  |  |  |
| I + II | Reference | - | Reference | - |
| III + IV | 2.615 (1.949 - 3.508) | <0.001 | 3.126 (2.257 - 4.329) | <0.001 |
| Surgery |  |  |  |  |
| No | Reference | - | Reference | - |
| Yes | 0.516 (0.381 - 0.699) | <0.001 | 0.554 (0.330 - 0.930) | <0.001 |
| Radiation |  |  |  |  |
| No | Reference | - | Reference |  |
| Yes | 0.496 (0.355 - 0.693) | <0.001 | 0.780 (0.449 - 1.355) | 0.378 |
| Chemotherapy |  |  |  |  |
| No | Reference | - | Reference | - |
| Yes | 0.703 (0.520 - 0.950) | 0.022 | 0.699 (0.482 - 1.012) | 0.058 |
| Differentiated grade |  |  |  |  |
| Well /Moderately differentiated | Reference | - | Reference | - |
| Poorly/Undifferentiated | 1.509 (0.938 - 2.427) | 0.090 | 2.209 (1.355 - 3.604) | 0.001 |
| Histology |  |  |  |  |
| AD | Reference | - | Reference | - |
| BSCC | 0.340 (0.233 - 0.495) | <0.001 | 0.387 (0.248 - 0.602) | <0.001 |
| Year of diagnosis |  |  |  |  |
| 2000-2009 | Reference | - | Reference | - |
| 2010-2019 | 0.369 (0.234 - 0.582) | <0.001 | 0.491 (0.286 - 0.843) | 0.010 |
| Marital status |  |  |  |  |
| Married | Reference | - | Reference | - |
| Unmarried^a^ | 1.319 (0.990 - 1.758) | 0.059 | 1.088 (0.808 - 1.465) | 0.579 |

BSCC, basaloid squamous cell carcinoma; AD, adenocarcinoma; T, tumor size; M, metastasis; N, node; HR, hazard ratio; CI, confidence interval; ^a^ Unmarried included single, divorced, widowed, and separated.
